# Promoter methylation of yes-associated protein (YAP1) gene in polycystic ovary syndrome

**DOI:** 10.1097/MD.0000000000005768

**Published:** 2017-01-13

**Authors:** Li-Le Jiang, Juan-Ke Xie, Jin-Quan Cui, Duo Wei, Bao-Li Yin, Ya-Nan Zhang, Yuan-Hui Chen, Xiao Han, Qian Wang, Cui-Lian Zhang

**Affiliations:** a Second Hospital Affiliated to Zhengzhou University, Zhengzhou, Henan Province; b Zhengzhou University, Zhengzhou, Henan Province; c Reproductive Medical Center, People's Hospital of Henan Province, Zhengzhou, Henan Province, P.R. China.

**Keywords:** in vitro fertilization embryo transfer, methylation, ovary granulosa cells, polycystic ovary syndrome, yes-associated protein 1

## Abstract

**Background:**

DNA methylation modification has been proved to influence the phenotype of polycystic ovary syndrome (PCOS). Genome-wide association studies (GWAS) demonstrate that yes-associated protein (YAP1) genetic sites are associated with PCOS. The study aims to detect the methylation status of YAP1 promoter in ovary granulosa cells (GCs) of PCOS patients and explore novel therapeutic targets for PCOS.

**Methods:**

Randomized controlled trial was applied and a total of 72 women were included in the study, including 36 cases of PCOS patients and 36 cases of health controls. Ovary GCs were extracted from in vitro fertilization embryo transfer. Methylation status of YAP1 promoter was detected by bisulfite sequencing PCR (BSP). Protein and mRNA expression of YAP1 were measured by western blotting and real-time quantitate PCR.

**Results:**

Overall methylation level of YAP1 promoter region from PCOS group was significantly lower than that from control group. CpG sites analysis revealed that 12 sites (−443, −431, −403, −371, −331, −120, −49, −5, +1, +9, +15, +22) were significantly hypomethylated in women with PCOS (*P* < 0.05). A significant upregulation of YAP1 mRNA and protein expression levels was observed. Testosterone concentration could alleviate the methylation status and demonstrate obvious dose–dependent relation.

**Conclusion:**

Our research achievements manifest that hypomethylation of YAP1 promoter promotes the YAP1 expression, which plays a key role in the pathogenesis and accelerate PCOS.

## 1 Introduction

The morbidity of polycystic ovary syndrome (PCOS) is gradually increasing and the etiology is diversiform, which makes it difficult to diagnose accurately and confidently. PCOS is characterized by amenorrhea, oligomenorrhea, polycystic ovary, infertility, and hyperandrogenemia.^[1,2]^ In addition, the PCOS women, even with regular menstrual cycles, have higher androgen and insulin levels than normal women. The clinical manifestations of PCOS have the characteristics of diversity and complexity, and large number of studies suggest that PCOS could be a combination of environmental factors and multiple genetic abnormalities. Presently, researches have not yet found the determinative gene which determines the pathogenesis and progression of PCOS.

Methylation modification of DNA is affected by plenty of environmental factors, which regulate the gene expression without altering the primary DNA sequence code.^[3]^ Methylation modification is a portion of epigenetic mechanism, which is heritable and invertible. Epigenetic includes DNA methylation, histone modifications, and small-noncoding microRNAs (miRNA).^[4]^ DNA methylation provides the most rigorous demonstration that epigenetic factors could obviously affect the biological characteristics. PCOS pathogenesis and progression are regulated by both genetic and epigenetic factors. That is, the genesis of PCOS is not only related to genetic alterations but also intimately connected with epigenetic changes. Although lots of factors have been proved play a vital role in PCOS progression by scholars and plentiful literatures have explained the mechanism, the DNA modification of ovary granulosa cell (GC) remains unclear.

Yes-associated protein (YAP1) is known as a pivotal transcriptional co-activator of Hippo pathway, which participates in regulation of tumor pathogenesis, for example, kidney, ovarian cancers, and breast.^[5,6]^ Further, YAP1 participates in many signaling pathways that regulate organic morphology, including ovary enlargement that is one of the major manifestations of PCOS. Genome-wide association studies (GWAS) showed that the single nucleotide polymorphisms of YAP1 (rs11225161, rs11225138, and rs11225166) were significantly different between PCOS and controls and it appears to be a new susceptibility gene for PCOS.^[7]^ YAP1 could combine with a series of transcription factors and act as a modulator through phosphorylation. The potential function of YAP1 on regulating PCOS is reported rarely. Our study aims to elucidate the difference of YAP1 promoter methylation between PCOS patients and health controls.

## 2 Materials and methods

### 2.1 Patients and granulosa cells collection

All the patients and samples were collected from the Reproductive Medical Center of People's Hospital of Henan Province and Zhengzhou University People's Hospital. The study has obtained approval and supervision of Ethics Committee of Henan Provincial People's Hospital of Zhengzhou University. All patients involved in this experiment have signed the informed consent and agreed the research purposes of clinical data and blood samples for our research. All patients enrolled in the study would be recorded general information, including age, height, weight, and gynecologic examination would be done by the same gynecologist. The BMI value of PCOS patients and healthy controls were in the normal range. Women in PCOS group were selected in strict accordance with the Revised 2003 Consensus on Rotterdam Diagnostic Criteria.^[8]^ Since there were other etiologies for hyperandrogenemia and ovulatory dysfunction, we rigorously selected the PCOS samples as exclusion criteria, including Cushing syndrome, thyroid disease, congenital, hyperprolactinemia, etc. PCOS patients were consistent with healthy controls in the indicators of age and BMI. Reproductive endocrine indexes were detected from peripheral blood at 2nd to 4th days of menstruation by chemiluminescence immunoassay through Cobas e411 (Roche, Mannheim, Germany), including follicle stimulating hormone (FSH), luteinizing hormone (LH), estradiol (E2), prolactin (PRL), progesterone, and testosterone.

GCs were collected from PCOS patients and controls that undergo their first in vitro fertilization embryo transfer. Collection and purification of GCs were mainly based on the density gradient centrifugation which was modified according to the experiment need. When the diameter of 3 or more follicles were above 17 mm, ovulation was induced by administering 4000 to 10,000 UI of human chorionic gonadotrophin (Pregnyl, Organon, Holland). After 35.5 to 37.5 hours, oocytes were obtained through transvaginal ultrasound guided aspiration. After oocyte was retrieved for insemination, the residual follicular fluid was collected and centrifuged as centrifugal separation processes.^[9]^ After centrifugation (200 × g, 10 minutes), precipitate was mixed with hyaluronidase (80 U/mL, SAGE, USA). After incubation (37 °C, 5% CO_2_) for 30 minutes, cell masses were pipetted and then underlayered with Ficoll-Paque Premium (GE Healthcare, Fairfield, CT) and were centrifuged (450 × g, 20 minutes) to separate. Finally, GCs were separated and washed with PBS and then stored at −80 °C for subsequent using.

### 2.2 DNA extraction and bisulfite sequencing PCR (BSP)

The extraction of DNA was operated through DNA extraction kit (TianGen, Beijing, China) from freezing and thawing GCs in accordance with the manufacturer instructions. Then, bisulfite conversion of DNA (1 μg) was manipulated with EZ DNA methylation-gold kit (Zymo, Orange, CA) according to the manufacturer's instructions. After bisulfite treated, the DNA was diluted into concentration of 20 ng/μL. Primers sequence of YAP1 was designed by the MethPrimer,^[10]^ which was as following: forward, 5′-TAATG TTGAAAATAATGGATTT TT-3′, reverse, 5′-CCCTT AACTACAAAAAATTCT TC-3′. Then, 1 μL DNA sample in 15 μL amplification reaction system, including 2 μL TaKaRa Taq Hot Start (TaKaRa, Dalian, China), was amplified with PCR. The amplification mode was touchdown PCR and the content was as following: an initial heat-start at 96 °C for 5 minutes, 10 cycles (including 95 °C for 20 seconds, 65 °C for 60 seconds, and 72 °C for 30 seconds for elongation), 30 cycles (including 95 °C for 20 seconds, 65 °C for 25 seconds, and 72 °C for 35 minutes for elongation). PCR products were purified by gel electrophoresis separation (Promega, Madison, WI). Purified PCR then was linked into PMD 18-T vectors (TaKaRa, Dalian, China). Primer of PMD 18-T plasmid was as following: AGCGG ATAACAATTTCACACAG GA-3′. Bigdye sequencing system was as following: 96 °C for 60 seconds, 25 cycles (including 96 °C for 10 seconds, 50 °C for 5 seconds, and 60 °C for 4 minutes). Plasmid DNA was extracted and sequenced using DNA analyzer (Applied Biosystems, Foster, CA).

### 2.3 Quantitative real-time PCR

Total RNA from GCs was isolated using Trizol (Invitrogen, Carlsbad, CA) according to the manufacturer's instructions. The RNA concentration was identified by ultraviolet spectrophotometer. The OD value at 260 to 280 nm was measured and the appropriate value was 1.8 to 2.0, which represented that the purity and concentration of RNA samples were up to standard. The reverse transcription reaction total system was 15 μL (containing 1 μg RNA). The reaction procedures were 42 °C for 60 minutes for elongation and 95 °C for 5 minutes for enzyme inactivation. Then, cDNA was synthesized and placed on ice for subsequent using. The preliminary primers were synthesized (Sangon Biotech, Shanghai, China). The PCR primer pairs were as following: YAP1, forward: 5′-GCTCA GATCCTTTCCTTA AC-3′, reverse: 5′-GCAGG GTGCTTTGGT TG-3′. β-actin, forward: 5′-CGACA GTATGCAGAAG GAG-3′, reverse: 5′-ACATC TGCTGGAAGGTG GA-3′. The PCR reaction condition was as following: a pre-denaturation was at 95 °C for 5 minutes and following by at 95 °C for 10 seconds, 60 °C for 30 seconds, 72 °C for 30 seconds and a final elongation step at 72 °C for 10 minutes. The amplification was 40 cycles. The dissolution curve was drawn at 65 to 95 °C. The internal reference was β-actin and the relative expression levels were expressed relative to it, which was calculated with the 2^−ΔΔCt^ method.

### 2.4 Western bloting

GCs were splited by RIPA Lysis Buffer System (Santa Cruz, Dallas, TX). The liquid was transferred into sterile EP tube by Eppendorf and placed on the ice for 15 minutes. Then, it was centrifuged 10,000 rpm for 15 minutes. Protein concentrations were measured by comparison with a known concentration of BCA. A mixture of equal parts of protein and sample buffer was subjected to SDS-PAGE. The separated protein then was transferred onto a PVDF membrane. Blocking buffer was prepared with TBST and 5% fat-free milk. The PVDF membrane was blocked in blocking buffer for 1 hours on shaking table at room temperature. Primary antibodies (Cell Signaling, Danvers, MA, no. 4912) were diluted (1:1000). β-actin acted as the internal reference. Primary antibodies were diluted by 2% fat-free milk. The PVDF membrane was incubated in the diluted primary antibodies 4 °C overnight and washed with TBST 3 times for 10 minutes. Then, the PVDF membrane was diluted in secondary antibody (Santa Cruz, Dallas, TX, sc-33732, 1:1000) 4 °C overnight. Finally, the PVDF membrane was washed again with TBST 3 times for 10 minutes. The PVDF membrane was displayed by Image Pro-Plus system (Media Cybernetics, Silver Spring, MD).

### 2.5 Statistical analysis

Data were expressed as a mean ± standard deviation and analyzed using SPSS software version 19.0 (SPSS, Chicago, IL). Categorical variables were described with counts and percentages. The differences between and within multiple groups were analyzed by one-way ANOVA or Student *t* test. *P* < 0.05 was considered to be statistically significant.

## 3 Results

### 3.1 General parameters of PCOS patients and controls

The clinical information and parameters are displayed as follows (Table 1). There is no significant difference on the age and BMI between PCOS patients group and controls group. Certain endocrine indicators, including LH and testosterone, are obvious higher in PCOS patients group than controls group and have significant statistical differences. Besides, FSH was obviously lower in PCOS group than controls, other indicators, such as prolactin, have no significant difference.

**Table 1:**
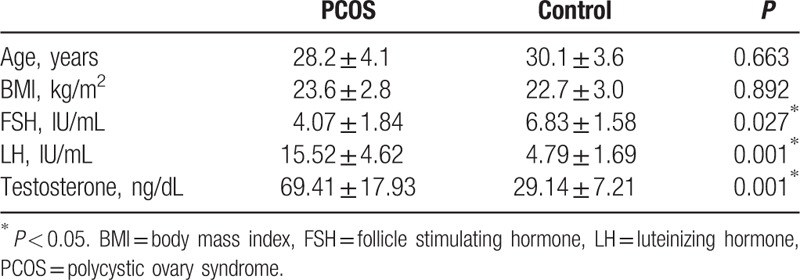
General parameters comparisons between PCOS and control group.

### 3.2 Methylation status of YAP1 promoter

**Figure 1:**
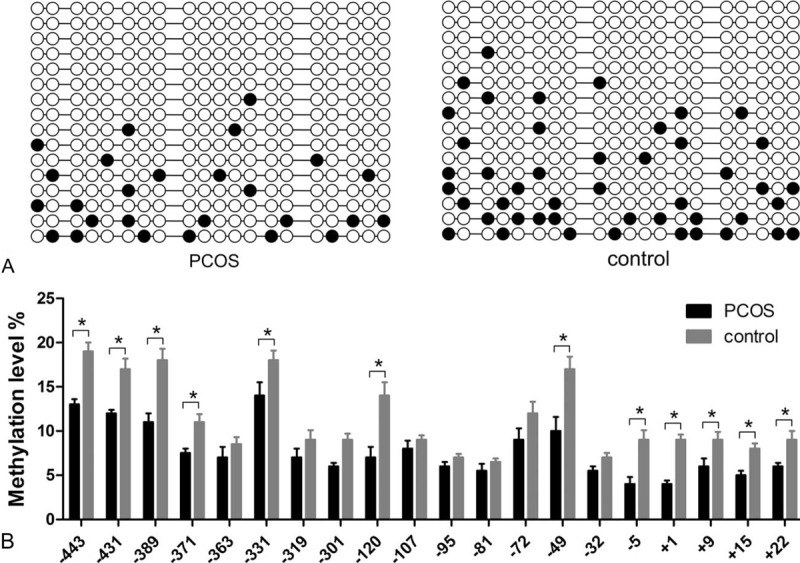
Methylation status of YAP1 was detected by bisulfite sequencing PCR. (A) Methylation profile of YAP1 in GCs. Each line represents an individual clone allele and each circle represents a CpG site. Filled circles represent for unmethylated and open circles represent for methylated CpG site. (B) Methylation status of CpG site in GCs and percentages represent the mean methylation level of each CpG site. ∗*P* < 0.05. GC = granulosa cell, PCR = polymerase chain reaction, YAP1 = yes-associated protein.

The methylation status of YAP1 promoter was detected by BSP and the sequencing was shown as follows (Fig. 1). It clearly showed that overall methylation level of YAP1 promoter region from PCOS group was significantly lower than that from control women. Sites analysis revealed that 12 CpG sites (−443, −431, 403, −371, −331, −120, −49, −5, +1, +9, +15, +22) were significantly hypomethylated in women with PCOS (*P* < 0.05).

### 3.3 Expression of YAP1 mRNA in PCOS and control group

**Figure 2:**
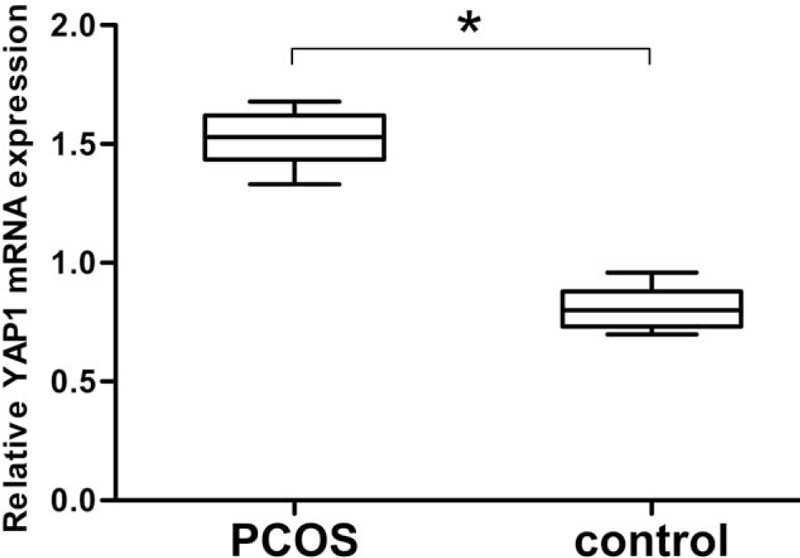
Real-time quantitative PCR analysis of YAP1 mRNA expression in the PCOS and control groups. The relative mRNA level of YAP1 was compared to that of β-actin. The ratio of YAP1/β-actin mRNA was 1.52 ± 0.28 in PCOS group, which was significantly higher than that in the control group (0.81 ± 0.08) (*P* < 0.05). Data represent means ± standard deviation. ∗*P* < 0.05 represents statistical difference between PCOS and control group. PCR = polymerase chain reaction, PCOS = polycystic ovary syndrome, YAP1 = yes-associated protein.

The mRNA expression of YAP1 was detected by real-time quantitative PCR. β-actin acted as internal reference and relative transcript level was shown as follows (Fig. 2). Results showed that the YAP1 mRNA expression level was significantly higher in PCOS patients than in controls (*P* < 0.05).

### 3.4 Protein expression of YAP1 in PCOS and control group

**Figure 3:**
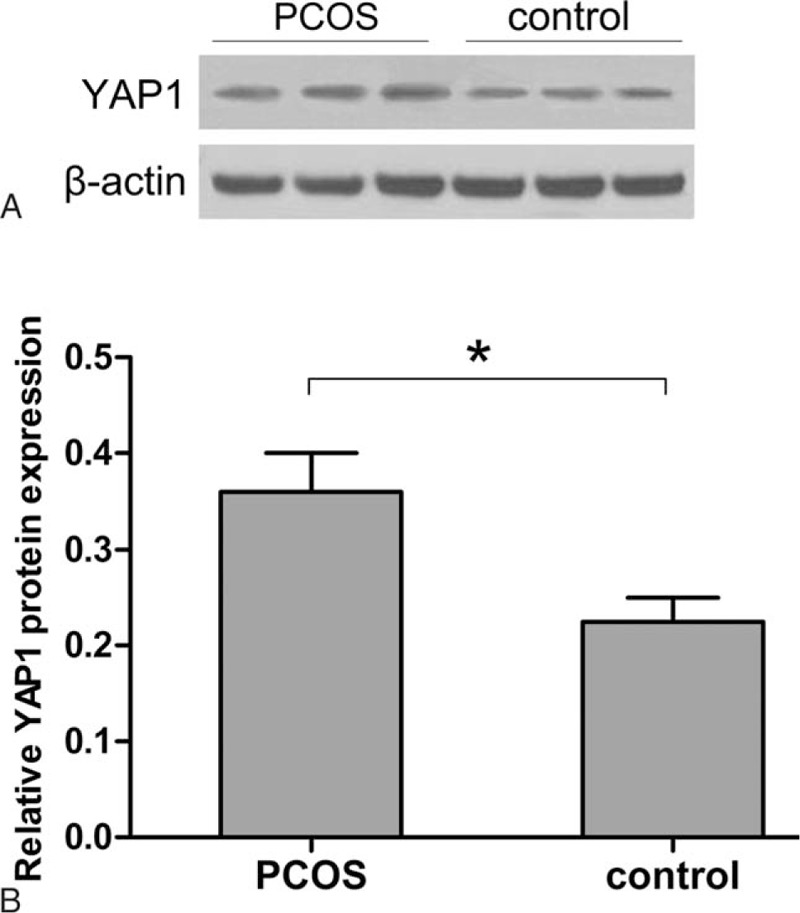
Western blotting analysis of YAP1 protein expression in the PCOS and control groups. (A) Randomized graphics were selected and exhibited to represent the total samples. β-actin acted as a loading control. (B) The relative protein expression of YAP1 in PCOS patients was relative to β-actin. YAP1 protein expression in PCOS group (0.374 ± 0.058) was statistically significantly higher than control group (0.241 ± 0.037). Data represent means ± standard deviation. ∗*P* < 0.05 represents statistical difference between PCOS and control group. PCOS = polycystic ovary syndrome, YAP1 = yes-associated protein.

Protein expression of YAP1 was detected by western blotting and shown as follows (Fig. 3). Protein imprinting and statistics results showed that the YAP1 protein expression level was significantly higher in PCOS patients than in controls (*P* < 0.05), which was in accordance with the tendency of YAP1 mRNA. Randomized graphics of western blotting were selected to represent the whole samples of PCOS and control group.

### 3.5 Different concentration of FSH, LH, and testosterone affect methylation level of YAP1

**Figure 4:**
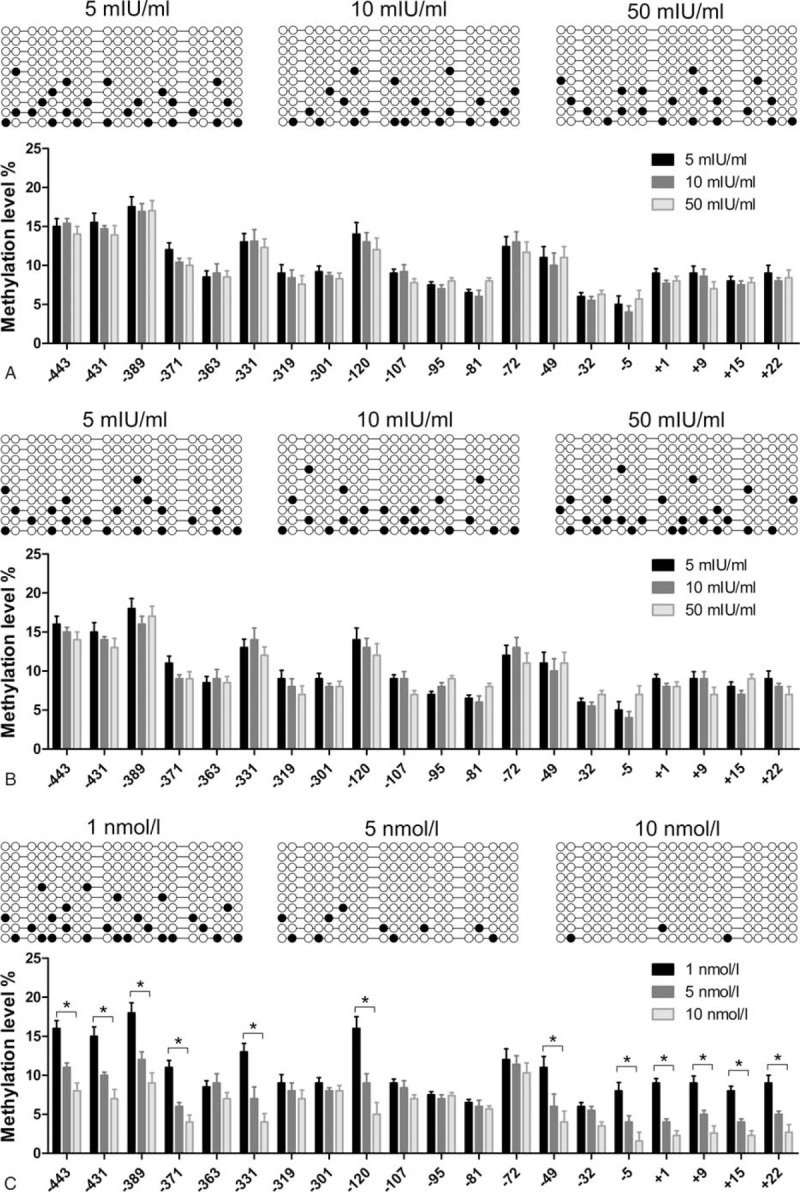
Different concentration of LH and testosterone act on the methylation status of YAP1. (A) BSP analysis showed the affection of FSH concentration (5, 10, and 50 mIU/mL) on the methylation status of YAP1 promoter. Physiological concentration was approximate 5 mIU/mL. (B) LH concentration (5, 10, and 50 mIU/mL) affect the methylation status of YAP1 promoter. Physiological concentration was approximate 5 mIU/mL. (C) Testosterone concentration (1, 5, and 10 nmol/L) affect the methylation status of YAP1 promoter. Physiological concentration was approximate 1 nmol/L. Data represent means ± standard deviation. ∗*P* < 0.05 represents statistical difference between PCOS and control group. BSP = bisulfite sequencing PCR, FSH = follicle stimulating hormone, LH = luteinizing hormone, PCR = polymerase chain reaction, YAP1 = yes-associated protein.

Anthropometric variables and endocrine parameters at the beginning of our study had revealed that LH and testosterone were significantly higher in PCOS group than that in control group (Table 1). In order to investigate the influence of FSH, LH, and testosterone on the methylation level of YAP1 promoter, we harvested GCs from follicular fluid of healthy women. The GCs, the same with above control samples group, were randomized divided into 9 groups and, respectively, given different concentration of FSH (5, 10, and 50 mIU/mL), LH (5, 10, and 50 mIU/mL), and testosterone (1, 5, and 10 nmol/L). YAP1 promoter methylation level through BSP and results were shown as follows (Fig. 4). The BSP analysis showed that FSH and LH concentration had a minimal effect on YAP1 methylation status. Nevertheless, testosterone concentration could alleviate the methylation status and demonstrate obvious dose–dependent relation.

## 4 Discussion

The application of GWAS provides a more comprehensive and pioneering achievements to research the susceptibility genes of PCOS.^[11,12]^ With human is entering a “post-GWAS” era, the primary task of epigenetics is to annotate the amass hereditary data that discovered by GWAS.^[13,14]^ The causal relationship and potential mechanisms between genetic marker and pathogenesis need to be investigated and sublimated. GWAS has showed that the single nucleotide polymorphisms of YAP1 are significantly different between PCOS and controls and it appears to be a new susceptibility gene for PCOS.^[7,15]^ Our study aims to detect the methylation status of YAP1 promoter in ovary GCs of PCOS patients and explores the epigenetic factors that motivate the pathogenesis.

A randomized controlled trial was adopted in our study. Patients who were diagnosed PCOS were enrolled into an experimental group, and an age-matched and BMI-matched control samples were arranged. Endocrine parameters examination shows that these 2 groups have statistic difference on the index of FSH, LH, and testosterone (Table 1). FSH, although the comparison is mildly significant, is lower in PCOS than control group. Combining with clinic manifestation and pathogeny, we conceive of the impact of hormonal in the inducement of PCOS, which will be verified in our subsequent experiments.

BSP, an efficient methylation detection means, was applied to sequence the methylation status of YAP1 promoter. Results reveal that overall methylation level of YAP1 promoter region from PCOS group is significantly lower than that from control women. Besides, there are 12 CpG sites (−443, −431, −403, −371, −331, −120, −49, −5, +1, +9, +15, +22), which are significantly hypomethylated in women with PCOS (Fig. 1). Furthermore, transcription and translation of YAP1 are elevated in women with PCOS compared with that in control women (Figs. 2 and 3). Protein and mRNA of YAP1 are synchronously increased in testing of western blotting and real-time quantitate PCR. The consequence is consistent with the decreased YAP1 methylation status associated with PCOS.

Our comparative study of different concentration of FSH, LH, and testosterone reveal that LH and FSH concentration variations do not have an obvious effect on YAP1 methylation status. Instead, increasingly concentration of testosterone could alleviate the methylation status and demonstrate obvious dose–dependent relation (Fig. 4). It has reach a consensus that overproduction of ovarian androgen is a pivotal pathologic feature of PCOS.^[16,17]^ Series of intracellular and extracellular signaling pathways involved in PCOS pathogenesis have been impressed by steroidogenesis and androgen.^[18,19]^ Prenatal androgen exposure and adolescent studies suggest that early androgen excess acts as an initiating factor of PCOS, but it still needs to confirm these hypothesis relying on convictive evidences.^[20]^ Scholars have reported long-term interventional studies that lower androgen levels in women with hyperandrogenism might protect against metabolic and cardiovascular comorbidities.^[18]^ The results suggest that methylation modification of YAP1 is an interaction factor that affects hormone level changes and PCOS. On the basis of our research, we realize that promoter methylation level of YAP1 could be affected by genetic factors and the hormone level of endocrine. The above findings connect the distribution of genetic markers with pathological physiology, which indirectly evidence that the intrauterine origin theory of PCOS.

Purpose of GWAS is to determine the candidate genes of disease and provide the possible locus of pathogenesis. The effect of locus determined by GWAS lasts a lifetime, but the epigenetic changes are dynamic during growth process.^[21,22]^ Studies have suggested that DNA methylation regulation exists in ovarian follicle growth and atresia.^[23]^ YAP1, known as a pivotal transcriptional co-activator of Hippo pathway, participates in many signaling pathways that regulate organic morphology, including ovary enlargement that is one of the major manifestations of PCOS.^[24,25]^ YAP1 could combine with a series of transcription factors and act as a modulator through phosphorylation. We examined genetic and epigenetic factors associating YAP1 with PCOS, which have representative significance. We attribute this limitation to our limited sample size and a larger samples research need to explore. However, the mechanisms underlying the regulation of YAP1 methylation remain to be fully elucidated.

The interaction relationship suggests that hypomethylation of YAP1 is a potential factor susceptibility to PCOS. It should be given more attention and investment to evaluate whether a causal relationship exists between YAP1 hypomethylation status and nosogenesis of PCOS.
